# Serological evidence of possible *Borrelia afzelii* lyme disease in Greece

**DOI:** 10.1016/j.nmni.2022.100978

**Published:** 2022-03-31

**Authors:** I. Karageorgou, M. Koutantou, I. Papadogiannaki, A. Voulgari-Kokota, S. Makka, E. Angelakis

**Affiliations:** Diagnostic Department and Public Health Laboratories, Hellenic Pasteur Institute, Athens, Greece

**Keywords:** *Borrelia afzelii*, Greece, Lyme borrelia, lyme disease

## Abstract

Suggestions that Lyme disease exists in Greece remain controversial and no study to date has definitively identified the presence of a *Borrelia* species that infects humans. We examined patients throughout Greece suspected for Lyme disease by enzyme-linked immunosorbent assay (ELISA) and by western blotting for *Borrelia burgdorferi* sensu lato species. We found one patient positive for *Borrelia burgdorferi* and two patients positive for *Borrelia afzelii* specific IgG antibodies. Both *B. afzelii* patients were suffering by neurological manifestations and had never traveled abroad. We provide serological evidence of two autochthonous Lyme disease cases in Greece, possibly caused by *B. afzelii*.

## Introduction

Lyme disease is caused by spirochetes within the *Borrelia burgdorferi* sensu lato species complex that are transmitted by specific *Ixodes* spp. Ticks [1]. In Europe, at least five species of Lyme borrelia (*Borrelia afzelii*, *Borrelia garinii*, *Borrelia burgdorferi*, *Borrelia spielmanii*, and *Borrelia bavariensis*) can cause the disease, leading to a wider variety of possible clinical manifestations [1]. The most common clinical manifestations in Europe are erythema migrans and Lyme neuroborreliosis, whereas *B. afzelii* is considered to be the predominant genospecies [1,2]. The incidence of Lyme disease has been increasing across the globe and it is estimated that more than 200,000 cases of Lyme disease occur in Europe each year [3]. Suggestions that Lyme disease exists in Greece remain controversial and no study to date has definitively identified the presence of a *Borrelia* spp. that infects humans. In addition, few serological studies have been conducted in animals from Greece, suggesting the possibility of *Borrelia* spp. presence in the country [[4], [5], [6]]. To our knowledge, physicians in Greece associate Lyme disease only with *B. burgdorferi* infection, as studies in the 1990s, based on enzyme-linked immunosorbent assay (ELISA), suggested the existence of *B. burgdorferi* [7]. However, since then, this hypothesis has never been confirmed and to date Lyme disease is not considered endemic. To determine this, we retrospectively examined for *B. burgdorferi* sensu lato species complex patients throughout Greece suspected for *B. burgdorferi* infection.

## Ethics statement

This study is based on routine diagnosis samples. All collected data were anonymized in standardized forms according to the Ethic and Scientific Committee of the Hellenic Pasteur Institute under registration number EIP-GDPR-E01.01.

## Materials

### Patients

We studied serum and blood samples obtained from hospitalized patients and outpatients throughout Greece that were sent to our laboratory from January to December 2019. As a referent laboratory, we routinely receive specimens from patients with suspected zoonotic infections throughout Greece. Inclusion criteria for participation in this study included age above 18 years and suspicion of zoonotic infection. Exclusion criteria was diagnosis by an agent other than *Borrelia* spp. Clinical data, medical history and complications during illness, habitat and environmental characteristics, presence of pets, livestock, or rodents, daily activities before symptom onset and travel during the previous one year were documented for patients with a positive western blotting (WB) result thanks to phone calls.

### Serological assays

A two-stage serodiagnostic (screening and confirmation) testing strategy was applied and firstly blood serum samples were screened by ELISA using the anti-*Borrelia* IgM and IgG ELISA (EUROIMMUN) kit to determine the presence of IgG and IgM *B. burgdorferi* sensu lato antibodies. ELISA results were classified as negative (< 16 RU/mL), equivocal (16–22 RU/ml) or positive (> 22 RU/mL) according to the manufacturer's instructions.

All ELISA-positive and equivocal serum samples were analyzed by WB using the anti- *Borrelia* spp. WB IgM and IgG (EUROIMMUN EUROLINE-RN-AT) for confirmation. The sum of the points attributed to each antigenic (OspA, OspC, p100, VlsE, p39, p58, p18, p41) band revealed on the strip according to their intensity was calculated and interpreted by the test strip analysis software, and scored as negative, positive, or equivocal. A sample was considered positive only if the positive or equivocal ELISA result in IgG or IgM was confirmed by a positive WB result.

### Molecular assays

Total genomic DNA was extracted (Biorobot EZ1 Workstation; QIAGEN, Courtaboeuf, France) from blood and serum samples of *Borrelia* spp. seropositive patients and were used as a template in a previously described real-time reverse transcription–PCR (RT-PCR) targeting the internal transcribed spacer (ITS) and a 16S rRNA gene sequence-based system, as previously described [8,9]. Two sets of negative controls (DNA of blood from a nonfebrile patient and sterile water) and a positive control (*B. recurrentis* DNA) were also analyzed in each run.

## Results

We totally studied 294 serum samples obtained from 294 different patients (56% females) and we identified seven (2%) samples positive and 21 (7%) samples with equivocal antibody index values by ELISA. All these samples were further tested by WB and a positive *B. burgdorferi* sensu lato result was obtained for three patients (Table 1). In addition, all blood samples remained negative by molecular assays for *Borrelia*. Detailed histories for each patient are described below.

**Table 1:** *B. burgdorferi* sensu lato species seropositive patients

Patient	ELISA (RU/mL)		Western blotting		Real time PCR	
	IgM	IgG	IgM	IgG	ITS	16S rRNA
1	18	77	Negative	*B. burgdorferi*	Negative	Negative
2	14	45	Negative	*B. afzelii*	Negative	Negative
3	11	95	Negative	*B. afzelii*	Negative	Negative

**Fig. 1:**
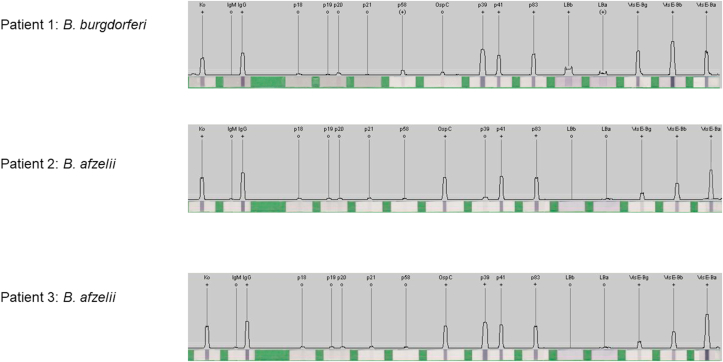
IgG Western blotting results for Borrelia burgdorferi sensu lato species complex positive patients.

The first patient was a 56-year-old male patient with an expanded erythema migrans on the right foot and epitrochlear adenopathy. WB was positive for *B. burgdorferi* specific IgG antibodies as was reactive for the p39, p41, p58, and p83 antigens and for *B. burgdorferi* VlsE specific antigen (Fig. 1). The patient reported a recent travel to Sweden, where he recalled a tick bite and a diagnosis of *B. burgdorferi* infection was given.

Patient 2, was a 43-year-old woman suffering by severe neurological manifestations, chronic encephalomyelitis, and encephalopathy. All tests for rheumatoid arthritis, systemic lupus erythematosus, myasthenia gravis, and other autoimmune diseases were negative for this patient. She presented specific IgG antibodies reactive for *B. afzelii* VlsE, OspC (p25), p41, and lipid *B. afzelii* antigens (Fig. 1). A diagnosis of possible *B. afzelii* infection was given but further investigation was not possible because she passed away. However, investigation after she passed away revealed that she presented a persistent erythema after an insect bite in the city of Athens one year before the symptoms' onset.

Patient 3 was a 35-year -old woman with neurological manifestations associated with persistent myalgia and arthritis of the large joints, especially the knee. She mentioned frequent outdoor activities, animal contacts and a cutaneous lesion after an insect bite close to the city of Larissa before the symptoms' onset. WB was reactive for IgG antibodies of *B. afzelii* VlsE, OspC (p25), p41, and lipid *B. afzelii* antigens (Fig. 1). A diagnosis of possible *B. afzelii* infection was given and a course of 200 mg of oral doxycycline once per day for three months was introduced.

## Discussion

We present two patients with autochthonous *B. afzelii* infection in Greece, based on their ELISA and WB serological reactivity. We believe that our serological results are accurate, as both our ELISA and WB assays have previously been evaluated and are routinely used for the diagnosis of Lyme disease. However, a limitation is that we did not detect *B. afzelii* by molecular assays. Both patients reported epidemiological risk factors and presented clinical manifestations that are commonly presented in patients suffering by Lyme neuroborreliosis or Lyme arthritis [1,2].

**Fig. 2:**
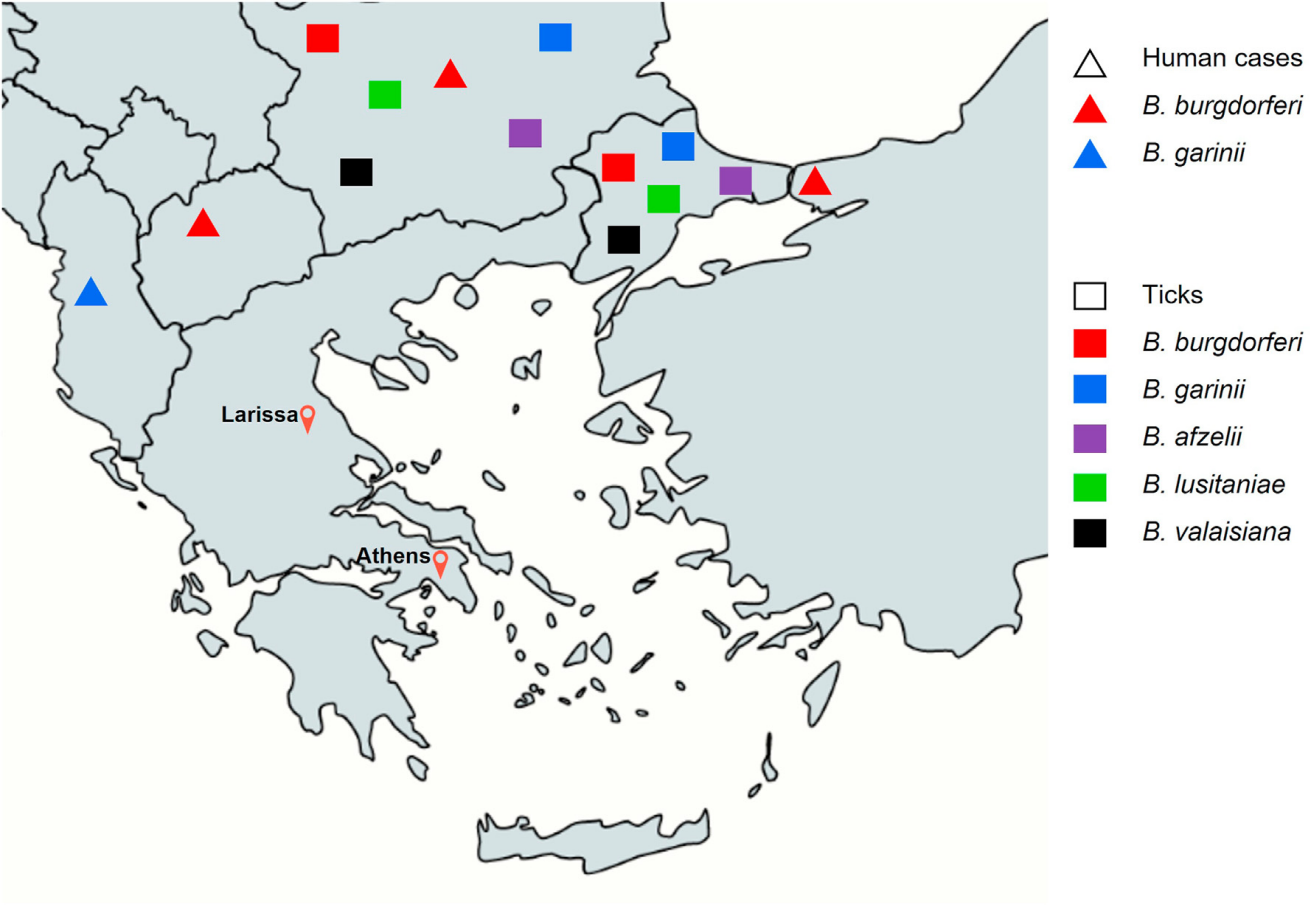
Human and tick presence of Borrelia burgdorferi sensu lato species to countries close to Greece.

The principal tick vector for *Borrelia* species in Europe is *Ixodes ricinus*. Although *I. ricinus* is prevalent in Greece, epidemiological studies have not detected *Borrelia* in it [10]. In contrast, *B. burgdorferi* sensu lato species complex is prevalent in ticks collected from countries close to Greece and *B. afzelii* was found to be the predominant species in *I. ricinus* ticks collected from Bulgaria [11]. Lyme disease pathogens, including *B. garinii* and *B. afzelii,* have also been detected in ticks collected in the Marmara Region of Turkey [[12], [13], [14]]. Additionally, Lyme disease has been known to be endemic in Albania, North Macedonia, Bulgaria and Turkey [[15], [16], [17]]; the reported incidence of Lyme borreliosis in Bulgaria is about 6/100,000 of the population [18], whereas in Turkey 75 cases of Lyme disease have been reported [19] (Fig. 2). However, the true incidence of the disease in these countries is probably much higher, because of the fact that the disease is often self-limited and mild cases remain unrecognized. Additionally, serological studies conducted in animals from Turkey and Bulgaria have reported seropositivity against *Borrelia burgdorferi* sensu lato in rodents [20,21], cats [22,23], dogs [[24], [25], [26], [27]], and horses [24,28,29]. In Greece, serological studies have reported a low seroprevalence (up to 2.2%) in dogs [5,6] but a significant seropositivity of 23.6% in sheep as reported in a study from 318 tick-infested sheep [4].

In conclusion, we demonstrate the possibility of the presence of *B. afzelii* in Greece. Therefore, we need to raise awareness of Lyme disease among healthcare providers and ensure that *B. afzelii,* and possibly also other Lyme borrelia species must be taken into account. The true underlying rate of Lyme disease in Greece remains unknown. The rarity of tick-borne diseases creates a surveillance and diagnostic challenge and the need to determine the true burden of Lyme disease in Greece in order to improve public health prevention messaging to healthcare providers and the public.

## Funding

None.

## Authors' contributions

**I.K.:** investigation**, M.K.:** Writing**, I.P.:** collect clinical data**, A.V.K.:** investigation**, S.M.:** Writing**, EA:** Writing, Supervision.

## Transparency declaration

There is no conflict of interest.
